# Finite Element Analysis of Maxillary Overdentures Supported by Two, Three, and Four Implants

**DOI:** 10.3390/bioengineering12121372

**Published:** 2025-12-17

**Authors:** Eduardo Borie, Eduardo Leal, Francisco Fernández-Gil, Renato Hunter, Benjamin Weber

**Affiliations:** 1 CICO Research Centre, Department of Integral Dentistry Adults, Universidad de La Frontera, Temuco 4811230, Chile; bejamin.weber@ufrontera.cl; 2 Departamento de Ingeniería Mecánica, Facultad de Ingeniería y Ciencias, Universidad de La Frontera, Temuco 4780000, Chile; eduardo.leal@ufrontera.cl (E.L.); francisco.fernandez@ufrontera.cl (F.F.-G.); renato.hunter@ufrontera.cl (R.H.)

**Keywords:** overdenture, finite element analysis, biomechanics, dental implants

## Abstract

This study aimed to evaluate, through finite element analysis (FEA), the biomechanical behavior of edentulous maxillary overdentures supported on 2, 3, and 4 implants with conometric connections, and to determine the minimum implant number that maintains stresses within physiological limits. A 3D finite element model of a resorbed edentulous maxilla was generated from CT images and processed in ANSYS v19.0. Subsequently, six models were simulated according to implant number (2, 3, or 4) and cortical bone thickness (0.5 mm or 1 mm). Conical connection implants and cobalt–chromium-reinforced overdentures with Equator attachments were modeled. Bilateral axial loads were applied and Von Mises equivalent stresses were calculated for implants and abutments, while maximum and minimum principal stresses were analyzed in bone. Results showed that the highest deformation and stress concentrations were observed in the two-implant models, with trabecular stresses ranging from 6.5 to 8.4 MPa, exceeding the 5 MPa safety threshold. In contrast, both three- and four-implant models maintained trabecular stresses below 3 MPa, while keeping cortical bone stresses within physiological limits. The three-implant tripod configuration demonstrated a comparable stress distribution to the four-implant models. From a biomechanical perspective, overdentures supported on four implants with 1 mm cortical thickness showed the most favorable performance. Nevertheless, the three-implant model represented a biomechanically acceptable and potentially cost-effective alternative, suggesting its viability as a simplified clinical option that warrants further investigation.

## 1. Introduction

Implant-supported rehabilitations have become an increasingly common prosthetic option, particularly among patients over 55 years [1]. Given the continuous growth in the geriatric population, the demand and clinical indications for dental implants are expected to rise steadily [2].

In this demographic group, the loss of masticatory function often leads to the avoidance of fibrous or hard foods, which are key sources of protein, fiber, vitamins, and minerals. This functional limitation highlights the importance of oral rehabilitation in older adults [3,4]. Fortunately, the introduction of dental implants has significantly improved both quality of life and prosthetic satisfaction among edentulous patients [5]. However, despite these advances, the high cost of implant-based treatments and their clinical requirements—such as the minimum number and distribution of implants needed for a successful rehabilitation—remain significant barriers. In the case of implant-retained maxillary overdentures, the literature frequently recommends at least four implants to achieve adequate function and prosthetic stability [6,7].

From a biomechanical standpoint, attachment type and implant distribution play a key role in load transfer to peri-implant bone. For instance, the use of rigid attachments to connect implants and overdentures has been shown to reduce stress on marginal bone, as demonstrated in the study by Chun et al. [8], in which overdentures retained by four implants with rigid attachments exhibited lower stress at the marginal bone level compared to other attachment systems.

In response to these limitations, various attachment systems have been developed to simplify treatment, reduce costs, and maintain satisfactory retention and stability—ultimately improving the quality of life of edentulous patients. In this regard, Locator attachments have demonstrated notable clinical and biomechanical advantages [9]. The most commonly used non-splinted attachments for connecting implants to removable overdentures include magnets, ball attachments, and Locator systems, with similar levels of stress reported at the implant level [8]. Clinically, the Locator attachment is often preferred due to its simplicity, stability, component durability, and interchangeable retentive inserts. It also allows for some degree of implant misalignment, making it one of the most frequently chosen options, particularly for maxillary overdentures, where it has shown superior clinical performance.

Although most studies on overdentures focus on the mandible, limited research is available regarding the biomechanical behavior of maxillary overdentures, particularly in relation to implant number, distribution, position, and connection type [10]. In the mandible, the use of two implants has shown favorable clinical outcomes, regardless of the attachment system used, especially in terms of patient satisfaction and retention [11,12]. In contrast, the gold standard in the maxilla remains the use of four implants. Nevertheless, a few studies have reported acceptable outcomes in terms of patient satisfaction using only two implants [13].

It is worth noting that increasing the number of implants complicates the achievement of proper parallelism, while using fewer implants tends to increase biomechanical stresses on the system’s components [14]. Using only two implants in the anterior maxilla—regardless of the attachment type—has been considered biomechanically unfavorable due to the higher stress concentrations involved [15]. It has been reported that stress distribution in palateless overdentures varies significantly depending on implant positioning, attachment type, and prosthetic design [16,17]. Despite this, there is limited information in the literature regarding the use of three implants in a tripod configuration, which may represent a biomechanically viable and clinically promising alternative, thus justifying further investigation.

Therefore, this study aimed to biomechanically evaluate, through 3D finite element analysis, the minimum number of implants that can be used in equidistantly distributed models without overloading the bone or implants. Despite being an in silico or computational study, the results may be relevant as a first step for future research that may lead to treatment alternatives with a smaller number of implants, simplifying the surgical procedure and reducing associated costs by at least 25%.

## 2. Materials and Methods

This study was conducted using a three-dimensional finite element model of an edentulous maxilla to compare stress distribution in the implants, prosthesis, and surrounding bone across the different models analyzed.

A digital image of an edentulous maxilla was obtained from a computed tomography database (CTI) and converted into a 3D model using InVesalius software (v3.0, CTI). The 3D model was exported to Rhinoceros software (v.6.0, McNeel North America, Seattle, WA, USA) for delimitation of the main anatomy. Maxilla resorption was manually modeled simulating a Class III condition according to the Cawood and Howell classification [18], ensuring adequate height and width. The further models consisted of trabecular bone surrounded by an external cortical layer of 0.5 or 1 mm in thickness.

Conical connection implants (Helix GM 3.75 × 10 mm, Neodent, Curitiba, Brazil) and overdenture attachments (Equator GM, Neodent, Curitiba, PR, Brazil) were sectioned using an electro-erosion machine (FW 1U, AgieCharmilles, GF Machining Solutions, Beijing, China). To enhance the accuracy of the subsequent 3D scan, the implant and abutment surfaces were coated with a penetrant ink (Spotcheck SKL-SP2 Penetrant, Magnaflux, Glenview, IL, USA) prior to scanning with a 3D scanner (Capture Mini, 3D Systems, RockHill, SC, USA). In the 3D model, the implants were positioned 1 mm below the bone crest and equidistantly distributed to support a metallic framework overdenture, which was also scanned. Also, a 1 mm thick mucosal layer was included in all models.

**Figure 1:**
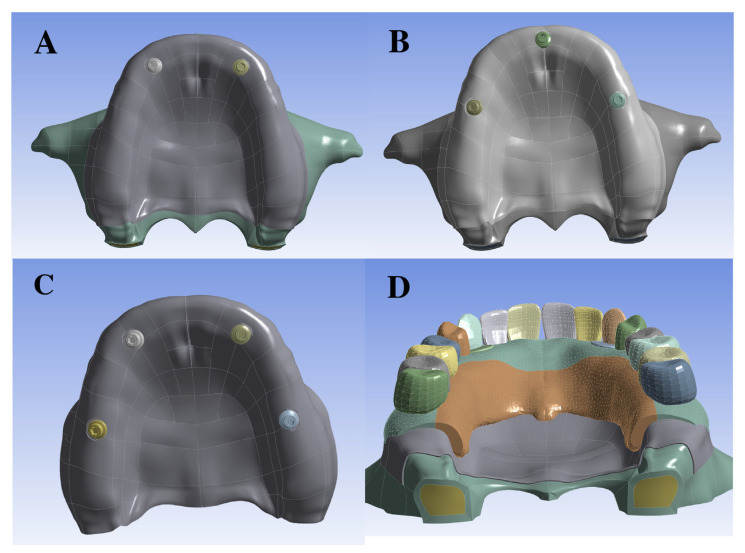
Implant distribution in the different models used in the study. (**A**) Models 1 and 2; (**B**) models 3 and 4; (**C**) models 5 and 6; (**D**) framework and prosthesis connected to implants.

The simulations were performed into six groups according to the implant number, bone type, and implant location within the maxilla, as follows:

(1) Two implants located at 13-23, considering a cortical bone with 0.5 mm thickness. (Figure 1A).
(2) Two implants located at 13-23, considering a cortical bone with 1 mm thickness.
(3) Three implants, with one located in the midline region and the others located at the second premolar level, considering a cortical bone with 0.5 mm thickness. (Figure 1B).
(4) Three implants, with one located in the midline region and the others located at the second premolar level, considering a cortical bone with 1 mm thickness.
(5) Four implants, two at the level of canines and two in the region of the first molar, considering a cortical bone with 0.5 mm thickness. (Figure 1C).
(6) Four implants, two at the level of canines and two in the region of the first molar, considering a cortical bone with 1 mm thickness.

**Figure 2:**
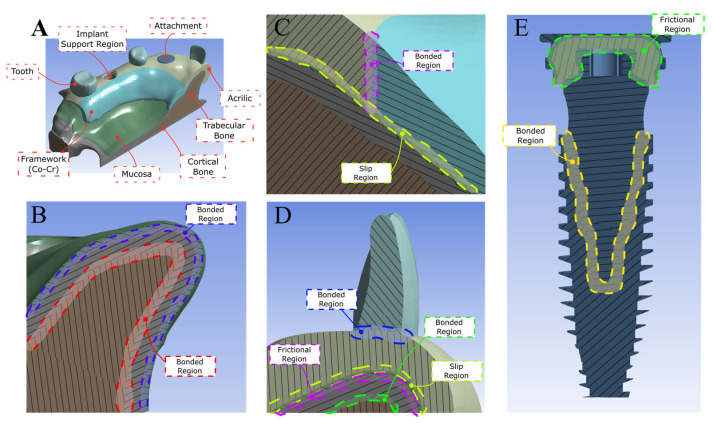
Scheme showing the contact types used in the models. (**A**) Three-dimensional CAD geometries used in the finite element model, including teeth, implant, attachment, acrylic structures, trabecular and cortical bone, mucosa, framework, and implant–attachment insertion regions. (**B**) Bonded contact interfaces: mucosa–cortical bone and cortical–trabecular bone. (**C**) Bonded interface between the acrylic component and the Co–Cr framework and slip contact between both and mucosa. (**D**) Bonded interface between the tooth and the acrylic structure; slip contact between the acrylic and the mucosa; and bonded contact between the mucosa and cortical bone. (**E**) Bonded contact between attachment and implant, simulating a conometric connection and the frictional region of attachment.

The implants were connected to a U-shaped cobalt–chromium metal framework overdenture coated with acrylic resin, which was attached to the Equator GM attachment system (Neodent, Curitiba, PR, Brazil) (Figure 1D). The 3D models were exported to the CAE software Ansys (v. 19.0, Ansys Inc., Canonsburg, PA, USA) for finite element simulation. All structures were assumed to be isotropic, homogeneous, and linearly elastic, with implants 100% osseointegrated. The trabecular bone–cortical, implant–bone, implant–abutment, and metal–base–acrylic interfaces were modeled as perfectly bonded. In contrast, the attachment system junction was simulated using a coefficient of friction of 0.2, requiring a nonlinear analysis. A no-separation contact condition was applied between the mucosa and framework to allow for interfacial sliding while preventing detachment (Figure 2). To represent the anatomical constraints imposed by the craniofacial structure, the superior and posterior surface of the maxilla was fixed using a fully constrained boundary condition. Material properties, including elastic modulus and Poisson’s ratio, were obtained from the literature (Table 1). The number of nodes and elements in all models ranged from 1,968,280 to 2,086,280 and from 1,283,132 to 1,344,126, respectively.

**Table 1:** Young modulus and Poisson coefficient of the materials used in the simulation.

Material	Young Modulus (GPa)	Poisson Coefficient	References
Titanium	115	0.35	[19]
Cortical bone	13.7	0.3	[20]
Trabecular bone	0.69	0.3	[20]
Mucosa	0.01	0.4	[21]
Framework (Co-Cr)	218	0.33	[21,22]
Nylon (*Equator* cap)	0.07	0.4	[21]
Acrylic resin	2.7	0.3	[21]

Then, bilateral loads of 75 N at the premolar level and 150 N at the first molar level were applied axially in the artificial molar fossa [21] to evaluate the biomechanical behavior of the prosthesis and to analyze, through finite element analysis, the stresses transmitted to the implant and the peri-implant bone.

## 3. Results

Stress distribution and total deformation in maxillary overdenture models are summarized in Table 2.

**Table 2:** Stress distribution and total deformation in maxillary overdenture models.

Model	Configuration	Maximum Principal Stress Cortical/Trabecular Bone (MPa)	Minimum Principal Stress Cortical/Trabecular Bone (MPa)	Von Mises Implant Stress (MPa)	Von Mises Abutment Stress (MPa)	Total Deformation (mm)
**1**	2 implants at canines; cortical 0.5 mm	7.1/8.4 *	−9/−14	250	60	0.165
**2**	2 implants at canines; cortical 1 mm	6.9/6.5 *	−8.7/−12	338	69	0.165
**3**	3 implants (2 premolars + 1 anterior); cortical 0.5 mm	10/2	−21/−2.6	170	54	0.130
**4**	3 implants (2 premolars + 1 anterior); cortical 1 mm	9.6/2	−20/−2.3	174	57	0.130
**5**	4 implants (2 molars + 2 canines); cortical 0.5 mm	6.1/2.9	−14/−1.9	136	40	0.130
**6**	4 implants (2 molars + 2 canines); cortical 1 mm	6.1/2.3	−11/−1.5	139	50	0.130

* Critical, over threshold.

**Figure 3:**
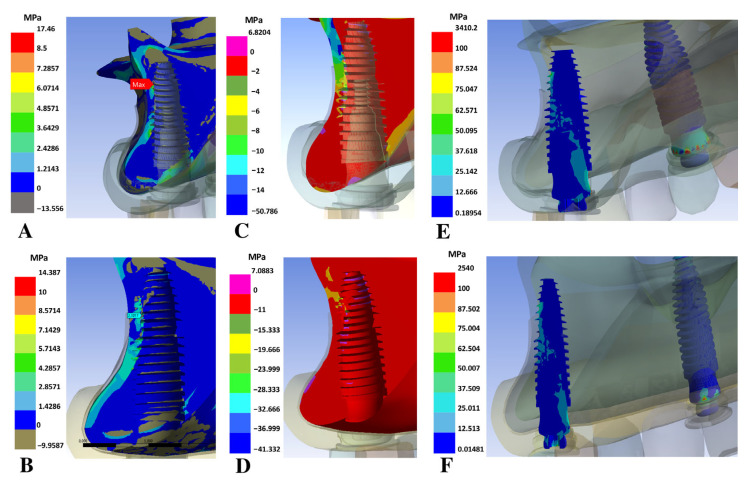
Stress comparison between models with two implants and four implants. (**A**) Maximum principal, minimum principal (**C**) and Von Mises (**E**) in model 2; (**B**) maximum principal, minimum principal (**D**) and Von Mises (**F**) in model 6.

**Figure 4:**
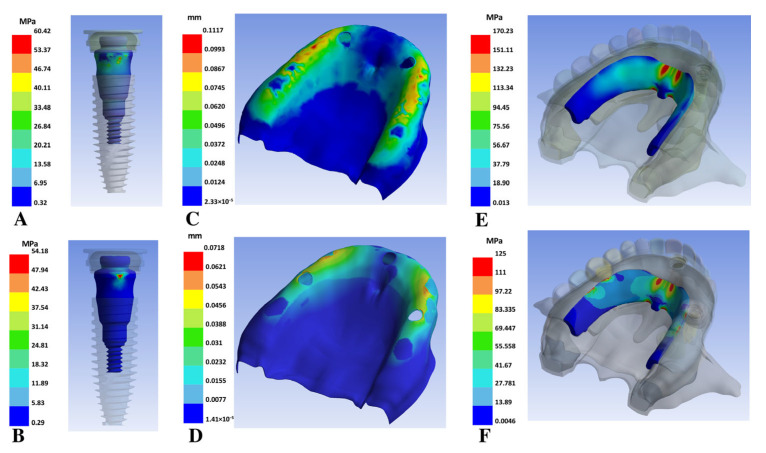
Stress comparison and total deformation between models with two implants and three implants in abutments and frameworks. (**A**) Von Mises stress in the attachment, (**C**) total mucosa deformation, and (**E**) Von Mises stress in the framework of model 1. (**B**) Von Mises stress in the attachment, (**D**) total mucosa deformation, and (**F**) Von Mises stress in the framework of model 3.

The highest total deformation values were observed in the models with overdenture supported on two implants. Similarly, the highest maximum principal stress values in the trabecular bone region were also found in the two-implant models (Figure 3A), ranging from 6.5 to 8.4 MPa, compared with values ≤ 2.9 MPa in the three- and four-implant models. The two-implant models exhibited the highest minimum principal stresses (Figure 3C) in the trabecular bone too, ranging from 12 to 14 MPa. Regarding the von Mises equivalent stress in the implants and abutments, the highest values were found in the two-implant models (Figure 3E and Figure 4A), with values decreasing progressively as the number of implants increased (Figure 3B,D,F and Figure 4B). The highest stress concentration was mainly observed in the anterior implants of models 3, 4, 5, and 6.

The total deformation of mucosa varied from 0.11 mm in both two-implant models to 0.07 mm in the three- and four-implant models (Figure 4C,D). The Von Mises equivalent stress in the framework of both two-implant models ranged between 170 and 180 MPa (Figure 4E), while in the three-implant models, it was 110–125 MPa (Figure 4F), and for the four-implant models, the lowest value was obtained (80–90 MPa).

## 4. Discussion

The purpose of this study was to evaluate the biomechanical behavior of metal–acrylic overdentures supported by two, three, and four implants using FEA.

It is well established that overdentures supported by four implants show survival rates exceeding 90% during the first 10 years [23,24]. The results of this study support this observation: the four-implant model with 1 mm cortical thickness (Model 6) showed the lowest stress concentration in the bone, as well as the lowest Von Mises stresses in the abutments and implants.

Conversely, the two-implant configurations generated trabecular stresses of 6.5–8.4 MPa, exceeding the 5 MPa safety limit for trabecular [25,26]. Such overload may initiate early fatigue damage in trabecular [22,27]. Therefore, models 1 and 2 are biomechanically deficient, exceeding trabecular tensile stress safety limits above 5 MPa, marking a significant risk for microfracture and subsequent bone resorption that may harm the osseointegration. These findings are consistent with those of Dimililer et al. [11], who also reported high stress concentration in bone supporting two-implant overdentures. Clinically, these results are in agreement with the clinical survival of overdentures using two implants, which has been reported to suffer a high rate of implant loss in the first year [24], with a rate of implant loss of between 25 and 30% in 5 years or more [28,29,30].

In contrast, the three- and four-implant models exhibited principal stresses in the trabecular bone ranging from 2 to 2.9 MPa, remaining consistently below the 5 MPa threshold [25,26]. The maximum and minimum stresses in the cortical bone reached up to 20 MPa, well within the physiological range (the tensile stress interval of cortical bone is 100 to 130 MPa, and the compressive stress interval is 170 to 190 MPa [25]) and without evidence of overload.

From a biomechanical standpoint, the three-implant configuration appears to be an effective and less complex alternative, maintaining stress levels within the physiological limits of the bone. However, as the number of implants increases, achieving accurate parallelism becomes more challenging. Previous studies related to overdentures supported by three maxillary implants are limited, but implant losses are observed that vary from 13.7% in 20 years [31] to 0% between 2 and 5 years or more [32,33]. Payne et al. [34] reported a survival rate of 92% during the first year in overdentures with implants using ball attachments with external hexagon narrow (3.3 mm) and tilted implants. All of the above studies include tilted implants, internal or external hexagon connections, and a metallic reinforcement only in the center of the acrylic overdenture. In our study, the implants were placed equidistant, considering a polygonal distribution, with conical connection implants, and with a metallic framework that supports the prosthesis with little palatal coverage. Therefore, all these characteristics could have contributed to the better stress distribution in the models with an overdenture using three implants. In this sense, the stress distribution over the implants and surrounding bone is strongly influenced by factors such as the metallic reinforcement of overdentures [35], the polygonal and equidistant implants distribution [36], their angulation [37], and the type of connection, with the preference being a straight position and conical connection [38].

The total mucosal strain values for all models were minimal and not considered relevant. Furthermore, the Von Mises stresses of the framework did not reach the fatigue limit considered in the literature (480 MPa [39]) in any of the models, while the stress concentration region was the same in all models.

Regarding cortical bone thickness, models with 1 mm of cortical bone showed a decrease in both maximum and minimum principal stress, which is in agreement with previous reports [40,41]. However, there was also an increase in the Von Mises stresses on the abutments and implants when compared to models with 0.5 mm cortical thickness. Nonetheless, the variation in the maximum principal stress, which corresponds to the stress at which bone is most fragile, was not significant, showing only a 3–4% decrease.

Although finite element analysis is commonly employed as an initial method for evaluating dental implants under various conditions, these results should be interpreted with caution. This study has several limitations, including the assumption of complete osseointegration of the implants, the simplification of trabecular bone as a homogeneous structure, and the use of static loading conditions. These assumptions were necessary due to computational constraints. Another limitation is that the implant positions did not follow the ideal clinical placement. To achieve maximal parallelism and ensure a standardized comparison across models, the implant angulations and locations were adjusted according to geometric constraints, which, in some cases, were different from the optimal anterior and posterior positions typically recommended in clinical practice. Furthermore, the analysis was performed on a single virtual anatomical model, which cannot adequately represent the variability of the population, particularly regarding bone density variations and trabecular architecture differences across individuals. The reliability of the finite element results depends largely on how accurately the models represent real anatomical and biomechanical conditions. Therefore, to validate these findings, further prospective clinical studies are required.

Recognizing the potential impact of the outcomes of this in silico investigation, the present work serves as an initial contribution to future research efforts focused on establishing the minimum number of implants required to ensure biomechanical integrity and concurrently optimize prosthetic functionality and patient satisfaction.

## 5. Conclusions

From a biomechanical point of view, the four-implant model with 1 mm cortical thickness demonstrated the most favorable outcomes in metal–acrylic overdentures. However, the model with three equidistant implants supporting a metal–acrylic overdenture showed acceptable stresses within the physiological ranges of the bone, representing a potential balance between biomechanical safety and cost/complexity reduction.

## Data Availability

The original contributions presented in this study are included in the article. Further inquiries can be directed to the corresponding author.
